# The relationship between the expression of Ki-67 and the prognosis of osteosarcoma

**DOI:** 10.1186/s12885-021-07880-y

**Published:** 2021-03-01

**Authors:** Ming Zeng, Jian Zhou, Lifang Wen, Yanshan Zhu, Yingquan Luo, Wanchun Wang

**Affiliations:** 1 Department of Orthopedics, The Second Xiangya Hospital, Central South University, 139 Renmin Middle Road, Changsha, 410011 China; 2 Department of Dermatology, Hunan Key Laboratory of Medical Epigenomes, The Second Xiangya Hospital, Central South University, Changsha, 410011 Hunan China; 3 Department of General Medicine, The Second Xiangya Hospital, Central South University, Changsha, 410011 Hunan China

**Keywords:** Ki-67, Meta-analysis, Prognosis, Clinicopathological features, Osteosarcoma, TCGA dataset

## Abstract

**Background:**

A number of studies have linked positive Ki-67 expression with the prognosis of osteosarcoma (OS) patients. However, the results have been conflicting. To address this controversy, we conducted an analysis using a meta-analysis and a TCGA dataset to estimate the value of Ki-67 expression in the prognosis of OS.

**Methods:**

A comprehensive search for relevant papers was conducted using NCBI PubMed, Embase, Springer, ISI Web of Knowledge, the Cochrane Library, and CNKI regardless of the publication year. The associations between Ki-67 expression and the clinical features and main prognostic outcomes of OS were measured. The TCGA dataset was also analyzed. The pooled odds ratio (OR) and its 95% confidential intervals (CIs) were utilized for statistical analysis.

**Results:**

Overall, a total of 12 studies with 500 cases were included, and the results indicated that the expression of Ki-67 was significantly associated with Enneking stage (OR = 6.88, 95% CI: 2.92–16.22, *p* < 0.05), distant metastasis (OR = 3.04, 95% CI: 1.51–6.12, *p* < 0.05) and overall survival (OR = 8.82, 95% CI: 4.68–16.65, *p* < 0.05) in OS patients. Additionally, we observed no significant heterogeneity among all retrieved studies. Associations between Ki-67 expression and overall survival and disease-free survival of sarcoma were confirmed using the TCGA and Kaplan-Meier plotter datasets.

**Conclusion:**

The present study strongly suggests that positive Ki-67 expression was associated with Enneking stage, distant metastasis, and overall survival of OS, and it may be used as a potential biomarker to predict prognosis and guide clinical therapy for OS.

**Supplementary Information:**

The online version contains supplementary material available at 10.1186/s12885-021-07880-y.

## Background

Osteosarcoma (OS) is a common malignant bone tumor that mainly originating from the metaphysis of long bones [1–3]. Many factors are responsible for prognosis, including demographics, sensitivity to chemotherapy and tumor size, site, and stage [4, 5]. In all age groups, up to 25% of OS patients have metastatic disease, occurring most frequently in the lung [6, 7]. The 5-year overall survival rate is significantly reduced in patients with metastases [8–10]. The increasing incidence of OS [11] has not only severely affected people’s health year by year but also increased social burden [12]. Despite substantial progress that has been made in the diagnosis and treatment of OS, the outcomes of patients remain unsatisfactory due to incomplete understanding of the mechanisms of the disease [13].

In recent years, although we have made great progress in the surgical treatment of osteosarcoma, the 5-year survival rate of osteosarcoma patients is still only approximately 60–70%, and the 5-year survival rate of osteosarcoma patients with lung metastasis is only approximately 10–20% [14]. In recent years, many prognostic biomarkers of osteosarcoma have been reported; for example, LRRC15 can be used as a prognostic biomarker and is an emerging therapeutic target [15]. Transferrin receptor-1 and VEGF may be potential prognostic factors [16]. Circulating miR-25-3p can be used as a novel diagnostic and prognostic biomarker [17]. Currently, the indicators for prognosis were mainly about location, tumor size, recurrence rate, clinical stage and distant metastasis. To measure these indicator was not precise and efficient. Therefore, it was really essential to identify a more representative biomarker for providing an effective prognosis for OS [4, 5].

The Ki-67 antigen was first identified by Gerdes and colleagues in 1983 with the use of a mouse monoclonal antibody. This name was derived from the German city of Kiel and the clone number on a 96-well plate [18]. The gene is located on chromosome 10q25-ter17, and the Ki-67 antigen is a nonhistone protein comprised of two isoforms that weigh 395 kDa and 345 kDa [19]. The protein is only present in the cells at G1, S, and G2 phases of the cell cycle and mitosis but is absent in resting cells at the G0 phase [20], which suggests its fundamental role in the regulation of cell proliferation. Indeed, overexpression of Ki-67 in cancer cells indicates its predictive potential in various neoplasms [21]. Scotlandi et al. reported that the expression of Ki-67 was related to the level of malignancy in bone tumors [22], while Gail et al. found that positive expression of Ki-67 staining was not significantly associated with the median relapse-free survival in Ewing’s sarcoma [23]. Although numerous clinical studies concerning the relationship between Ki-67 and OS have been published in recent years [24–30], there is still a great degree of inconsistency among studies. Therefore, the role of Ki-67 in the prognosis of osteosarcoma is still uncertain. The main purpose of a meta-analysis is to reflect the results of previous studies more objectively and comprehensively to draw conclusions closer to the truth. In the present study, we performed a meta-analysis to assess the prognostic value of Ki-67 expression in OS patients. We present the following article in accordance with the PRISMA reporting checklist.

## Methods

### Search strategy and study selection

A systematic literature search of NCBI PubMed, Embase, Springer, ISI Web of Knowledge, the Cochrane Library, and CNKI was conducted to identify all relevant articles without language and publication year limitations. The ending date of literature collection was January 2020. Three search terms, “Ki-67”, “osteosarcoma”, and “prognosis” were combined with the Boolean operator “and”. The search strategies were as follows: (1) marker of proliferation Ki-67 or MKI67 or Ki-67 or MIB-1 or Mindbomb E3 ubiquitin protein ligase 1; (2) osteogenic tumor or osteosarcoma; and (3) prognostic or prognosis or survival. Two authors searched the papers independently and excluded irrelevant papers. The reference part of retrieved articles was screened in case of missing the original search.

### Inclusion and exclusion criteria

The inclusion criteria were as follows: (1) articles were published in Chinese or English; (2) papers contained original research on humans; (3) the full text was available and sufficient information was provided for estimation; (4) pathological results (i.e., the gold standard) were used for the diagnosis of OS; and (5) Ki-67 in OS was measured with immunohistochemistry.

The exclusion criteria were as follows: (1) Repeated researches. (2). Reports without survival outcome.(3) Wrong article types without original data. (4) No cut-off value for Ki-67 indicated in the articles. (5) No biopsy for diagnosis.

### Data extraction

Based on the exclusion and inclusion criteria, two investigators independently evaluated the eligibility of all retrieved papers. Discrepancies between the 2 investigators were resolved by discussion with a third investigator to reach consensus. Relevant information was extracted from the included studies, including Ki-67 assessment methods, case number, sex, median age, publication date, research country inclusion period, and first author. We contacted the corresponding author when further information was needed. If we did not receive any replies after three emails, we excluded the study.

### Assessment of included studies

The Newcastle-Ottawa Scale (NOS) [31] was used to evaluate the quality of all the published papers. The included studies were divided into three categories according to the score: 0–3, 4–5, and 6–8 were considered low quality, medium quality, and high quality, respectively.

### Assessment of prognosis in the TCGA dataset and Kaplan-Meier plotter dataset

Gene Expression Profiling Interactive Analysis (GEPIA) (http://gepia.cancer-pku.cn/) was adopted for further evaluation of the differential expression pattern of Ki-67 between normal samples and cancer for various tumors in the TCGA dataset. Additionally, the associations between Ki-67 and overall survival and disease-free survival were plotted as Kaplan-Meier curves using the TCGA dataset and Kaplan-Meier plotter dataset (https://kmplot.com/analysis/index.php?p=service).

### Statistical analysis

The OR and its 95% CI were calculated to evaluate the relationship between the incidence of Ki-67 overexpression and the prognosis of OS patients. The chi-square test was conducted to estimate heterogeneity [32]. A random effect model was used when there was significant heterogeneity (*p* < 0.10 and I-squared > 50%) [33]. Otherwise, (*p* > 0.10 and I-squared ≤50%), we chose a fixed-effect model [34]. Sensitivity analysis was conducted by sequentially omitting one of the studies to identify the underlying influence of the individual studies and assess the stability of the results. All the data analyses were conducted using STATA 12.0 software (StataCorp LP, College Station, TX, USA). Significance of a two-tailed test was set at *p* < 0.05.

## Results

### Search results

**Fig. 1:**
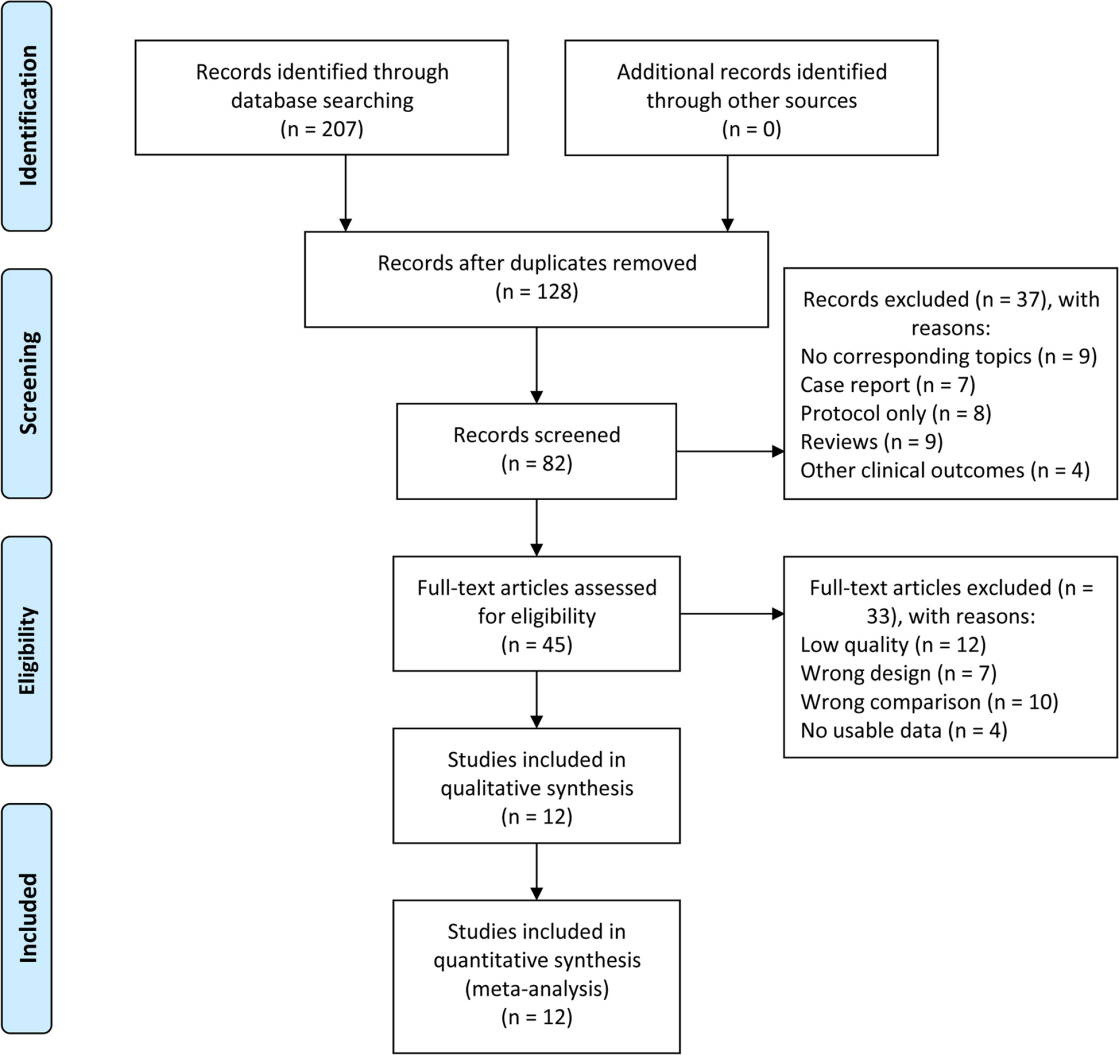
Flow diagram of paper selection

A total of 207 articles were retrieved in the primary search, and 128 reports remained after removing duplicated publications. Then, 46 papers were deleted after further screening, and 82 publications remained. Among them, 37 articles were excluded because they were not related to this topic. After further assessment of the 45 potentially eligible articles, 33 were excluded because of the lack of clinical studies. Finally, 12 relevant articles [24–30, 35–39] published from 1998 to 2018 were adopted in the presented meta-analysis (Fig. 1).

### Study characteristics

The main features of the 12 remaining studies containing 500 OS patients are listed in Table 1. All the patients involved in the eligible articles were Asian. Immunohistochemistry (IHC) detection methods were used in these studies. Among all these articles, one study lacked data on patient gender, two did not provide the median age, and one paper was missing information on the inclusion period.

**Table 1:** Features of 12 articles included in this meta-analysis

REF	First author	Year	Cases	Gender (M/F)	Median age	Inclusion period	Method	ki-67 cut-off	Ethnicity	NOS score
1	Wang et al.	2018	50	28/22	24.15	2015–2017	IHC	A1*B1 > 3	Asian	8
2	Fu et al.	2017	20	9/11	11.27	2008–2012	IHC	A1 ≥ 1	Asian	8
3	Li et al.	2017	21	11/10	24.33	2011–2013	IHC	A1 ≥ 1	Asian	8
4	Lin et al.	2014	55	31/24	17.42	2004–2011	IHC	A1*B2 > 3	Asian	7
5	Li et al.	2014	94	59/35	–	2006–2010	IHC	B3 ≥ 2	Asian	7
6	Matsumoto et al.	2013	29	19/10	19.00	1978–2007	IHC	A1 ≥ 1	Asian	8
7	Junior et al	2003	25	13/12	29.00	1958–2001	IHC	A1 ≥ 1	Asian	8
8	Xu et al.	2003	30	16/14	19.83	–	IHC	A3 ≥ 2	Asian	7
9	Zhang et al.	2003	30	16/14	–	1994–1999	IHC	A3 ≥ 2	Asian	7
10	Peng et al.	2002	62	–	17.00	1995–2001	IHC	A1 ≥ 1	Asian	8
11	Zhang et al.	2001	45	28/17	17.60	1993–1998	IHC	A1 ≥ 1	Asian	8
12	Liao et al.	1998	39	26/13	20.10	1992–1995	IHC	A1 ≥ 1	Asian	7

**A: Positive cell percentage:** A1: scored 0 (< 5%), 1 (6–25%), 2 (26–50%), 3 (> 50%); A2: scored 1 (< 25%), 2 (26–50%), 3 (51–75%), 4 (> 75%); A3: scored 0 (< 5%), 1 (5–20%), 2 (> 20%)

**B: Staining intensity:** B1: scored 0 (light yellow), 1 (brownish yellow), 2 (brown); B2: scored 0 (absence of staining), 1 (weak staining), 2 (middle staining), 3 (strong staining); B3: scored 0 (absence of staining), 1 (light yellow), 2 (brownish yellow), 3 (brown)

### Qualitative assessment

The quality of eligible studies was evaluated by NOS. A higher score (0–9) represents better methodology. The NOS scores of these 12 studies ranged from 7 to 8 (average score = 7.58) (Table 1); further information is provided in Supplementary Table 1.

### Relationship between Ki-67 and OS

**Fig. 2:**
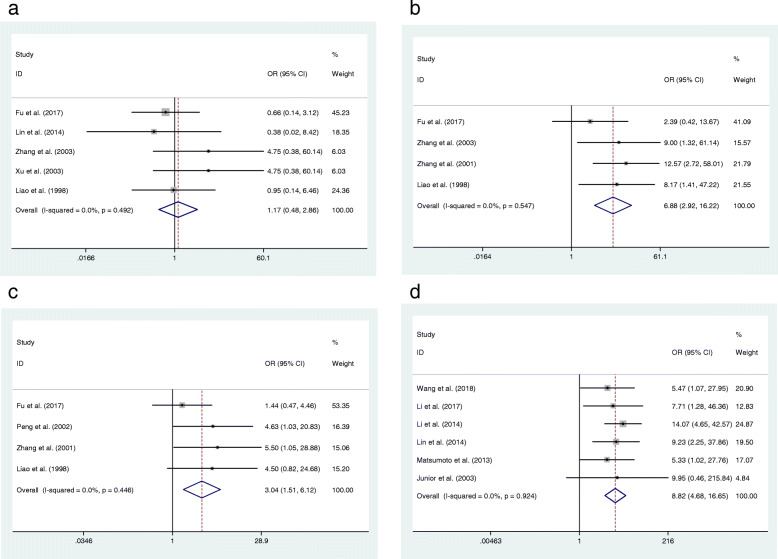
Forest plot of the association between Ki-67 expression and OS. **a**: classification, **b**: enneking stage, **c**: metastasis, **d**: overall survival

In the present study, we assessed the relationship of Ki-67 expression and clinicopathological features or prognosis of OS. No significant heterogeneity among those eligible studies was found (I^2^ < 50%), and a fixed-effect model was applied to combine the results of individual studies. The relation was evaluated by the pooled OR with its 95% CI. The results of the meta-analysis indicated that overexpression of Ki-67 in OS was associated with the Enneking stage of tumors (OR = 6.88, 95% CI: 2.92–16.22, *p* < 0.05) (Fig. 2b). Moreover, Ki-67 was shown to be correlated with distant metastasis (OR = 3.04, 95% CI: 1.51–6.12, *p* < 0.05) (Fig. 2c). Additionally, six papers (Table 2) were enrolled to explore the association between expression of Ki-67 and over survival of OS and we found that there was an association between the positive expression of Ki-67 and the 5-year overall survival of OS (OR = 8.82, 95% CI: 4.68–16.65, *p* < 0.05) (Fig. 2d). However, Ki-67-positive expression was confirmed to be irrelevant to OS classification (OR = 1.17, 95% CI: 0.48–2.86, *p* > 0.05) (Fig. 2a).

**Table 2:** Features of papers for survival of ki-67 on osteosarcoma

No.	Trial	Year	High expression		Low expression		Outcomes	Follow-up (month)
			Death	5-year survival	Death	5-year survival		
1	Wang et al.	2018	13	19	2	16	over survival	60
2	Li et al.	2017	12	7	2	9	over survival	60
3	Li et al.	2014	26	17	5	46	over survival	60
4	Lin et al.	2014	24	13	3	15	over survival	60
5	Matsumoto et al.	2013	12	3	6	8	over survival	60
6	Junior et al.	2003	13	9	0	3	over survival	60

### Sensitivity analysis

**Fig. 3:**
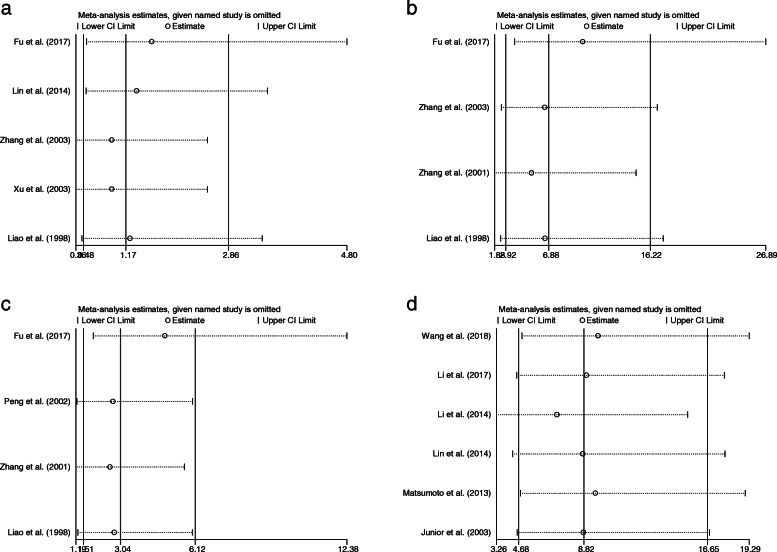
Sensitivity analysis indicating the association between Ki-67 expression and OS. **a**: classification, **b**: enneking stage, **c**: metastasis, **d**: overall survival

A sensitivity analysis was conducted to assess the stability of the results of the meta-analysis. The heterogeneity did not change significantly when omitting one of the combined papers. Therefore, we could conclude that the analysis results did not rely on individual studies, and the conclusion was credible (Fig. 3).

### Association between Ki-67 and OS prognostic features in the TCGA dataset

**Fig. 4:**
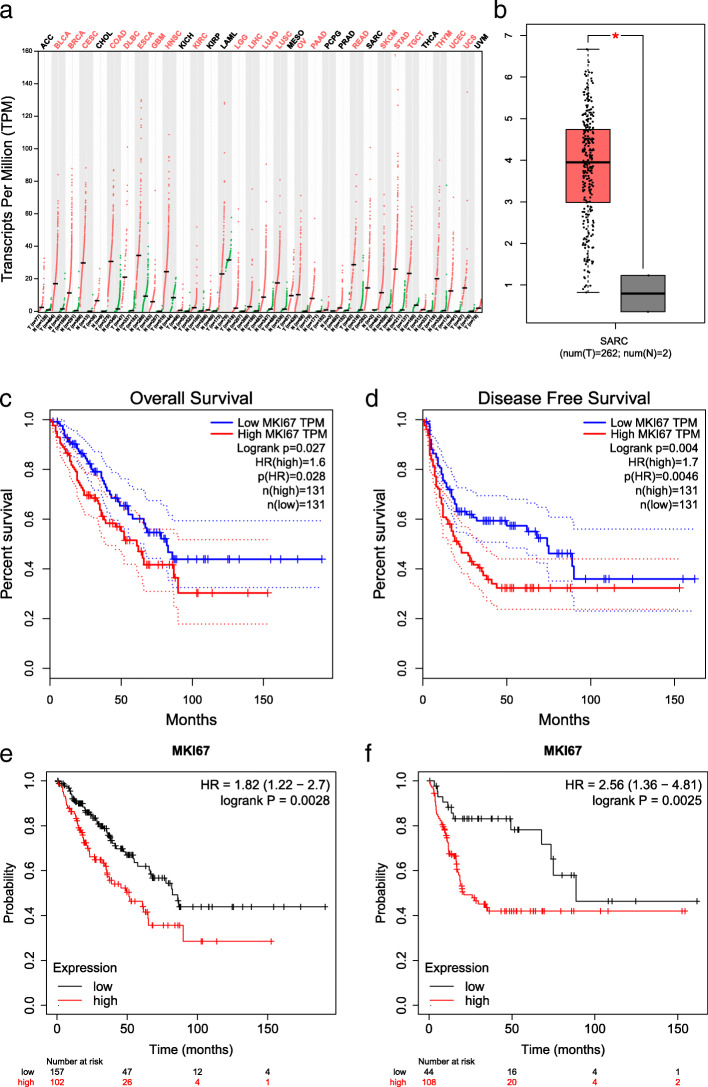
Analysis for the expression and prognosis of Ki-67 in cancers. **a**: differential expression of Ki-67 in various cancers, **b**: differential expression of Ki-67 in sarcoma, **c**-**f**: association between Ki-67 and over survival (**c** and **e**), disease free survival (**d**) and recurrence free survival (**f**) in sarcoma

The TCGA pan-cancer dataset and Kaplan-Meier plotter dataset were used to further validate the relationship between Ki-67 positive expression and prognostic features of OS. The results indicated that Ki-67 was significantly upregulated in many cancers including sarcoma (SARC), stomach adenocarcinoma (STAD), lung adenocarcinoma (LUAD), lung squamous cell carcinoma (LUSC), colon adenocarcinoma (COAD) and liver hepatocellular carcinoma (LIHC) (Fig. 4a). In addition, we found that Ki-67 was significantly upregulated in sarcoma (SARC) (Fig. 4b). Furthermore, the relationship between Ki-67 expression and OS and DFS was shown by Kaplan-Meier curves. The results of the TCGA dataset indicated that Ki-67 positive expression was significantly associated with overall survival (*p* < 0.05, HR = 1.6) (Fig. 4c) and disease-free survival (*p* < 0.05, HR = 1.7) (Fig. 4d). The results of the Kaplan-Meier plotter showed that Ki-67 positive expression was significantly related to overall survival (*p* < 0.05, HR = 1.8) (Fig. 4e) and recurrence-free survival (*p* < 0.05, HR = 2.5) (Fig. 4f) in sarcoma.

## Discussion

OS is a primary malignant bone tumor among young adults [40]. OS incidence has an age-specific bimodal pattern: the highest incidence occurs in adolescence and among those older than age 60 [41]. The metaphyses of long bones are the most common sites of OS in young patients. OS incidence is similar in childhood and adolescence and varies little by sex and race worldwide [42–44]. OS is characterized by easy metastasis and recurrence. Individuals with metastatic disease tend to have much poorer outcomes and lower 5-year survival. Although chemotherapy has improved the overall survival rate, the fatality rate is still high. The 5-year survival was approximately 60% for OS patients without visible metastases at the time of diagnosis but was reduced to 15% if tumor metastasis occurred in patients. Therapies for OS have not changed significantly over the past 3 decades, and this bottleneck needs to be overcome as soon as possible. Incremental progress is possible in OS therapies if novel prognostic biomarkers are included in clinical trials [45–47].

Ki-67, also called MKI-67, is expressed only in actively proliferating cells and is a proliferation-related nuclear antigen. Due to the overexpression of Ki-67 in cancer cells, it has been proposed as a prognostic biomarker of cancer [21]. Numerous retrospective studies have reported on the relationship between Ki-67 expression and the prognosis of prostate cancer [48], renal cell carcinoma [49] and several other cancers [50, 51].

In this study, we focused on the predictive effect of Ki-67 positive expression on the prognosis of OS. Li and Zhang suggested that the level of Ki-67 was related to the prognosis of patients with OS [25], but Junior and colleagues were not able to find a correlation between the marker and the prognosis, possibly because of the small number of cases [37]. Although many studies have suggested that Ki-67 is useful for predicting tumor grade [25, 26, 29], there are still other investigators who have drawn the opposite conclusion [26]. The relationship between Ki-67 and metastasis is also controversial [28, 29, 39, 52]. Considering the conflicting results, we investigated the correlation of Ki-67 expression with the clinicopathologic features and prognosis of OS using meta-analysis. The results identified Ki-67 as a predictive marker for reduced 5-year overall survival (OR = 8.82, 95% CI: 4.68–16.65, *p* < 0.05) in patients with OS. It can also be used as an independent risk factor for distant metastasis (OR = 3.04, 95% CI: 1.51–6.12, *p* < 0.05). Furthermore, the Ki-67 index indicated surgical Enneking staging of OS (OR = 6.88, 95% CI: 2.92–16.22, *p* < 0.05), while positive expression of Ki-67 was not related to OS classification (OR = 1.17, 95% CI: 0.48–2.86, *p* > 0.05). Additionally, the relationship between Ki-67 and worse survival outcomes in sarcoma was further confirmed using the TCGA dataset and Kaplan-Meier plotter dataset. In summary, the present study revealed that Ki-67 was a valuable marker of OS clinicopathological features and prognosis.

There are several limitations of our study into consideration. First, potential publication bias may exist as articles with positive results are easier to publish, which may influence the overall results. Second, the language of the included documents was limited to English and Chinese, which may have also had an impact on the accuracy of the results. Third, the results from these dataset were about sarcoma rather than osteosarcoma, which may affect the validation for this meta-analysis. Fourth, all included patients were from Asian, so the ethnicity may also attribute to potential bias. Last but not least, although all of the patients included were diagnosed with the gold standard (the pathological result), the pathological stage of each patient may also have had an effect on the outcome to some extent. Further multicenter studies with larger sample sizes are needed to reveal the internal correlation of Ki-67 and its predictive role in clinical work; this will decrease sample biases and minimize unavoidable random errors in the meta-analysis process.

## Conclusion

In the present study, a meta-analysis was performed to evaluate the relationship between Ki-67 expression and the clinicopathological features and prognosis of OS. Our study showed that Ki-67 positivity was related to the OS Enneking stage and distant metastasis. The results of the meta-analysis and TCGA dataset also indicated a dismal 5-year overall survival for OS patients with Ki-67 expression. Ki-67 may be a valuable biomarker for OS prognosis.

## Supplementary Information

**Additional file 1 Table S1.** Qualitative assessment of included study.

## Data Availability

The datasets used and/or analyzed during the current study are available from the corresponding author upon reasonable request.

## Abbreviations

OS: osteosarcoma
PIP3: phosphatidylinositol 3,4,5-trisphosphate
CDK2: cyclin-dependent kinases 2
CNKI: China National Knowledge Internet database
CBM: Chinese Biological Medical Database
NOS: Newcastle-Ottawa quality assessment scale
OR: odds ratio
95% CI: 95% confidence interval
TCGA: The Cancer Genome Atlas.
