# Supplementary material for: The relationship between the expression of Ki-67 and the prognosis of osteosarcoma
Source: BMC Cancer. 2021 Mar 1;21:210. doi: 10.1186/s12885-021-07880-y (PMC7923819; doi:10.1186/s12885-021-07880-y)
Supplement: Supplementary file 1 — Additional file 1 Table S1. Qualitative assessment of included study. [file 12885_2021_7880_MOESM1_ESM.docx]

**Supplementary table 1. Qualitative assessment of included study.**

| Column | Entries | Study | | | | | | | | | | | |
| --- | --- | --- | --- | --- | --- | --- | --- | --- | --- | --- | --- | --- | --- |
|  |  | 1 | 2 | 3 | 4 | 5 | 6 | 7 | 8 | 9 | 10 | 11 | 12 |
| Section | Is the definition adequate | ☆ | ☆ | ☆ | ☆ | ☆ | ☆ | ☆ | ☆ | ☆ | ☆ | ☆ | ☆ |
|  | Representativeness of the cases | ☆ | ☆ | ☆ | ☆ | ☆ | ☆ | ☆ | ☆ | ☆ | ☆ | ☆ | ☆ |
|  | Selection of controls |  |  |  |  |  |  |  |  |  |  |  |  |
|  | Definition of controls | ☆ | ☆ | ☆ | ☆ | ☆ | ☆ | ☆ | ☆ | ☆ | ☆ | ☆ | ☆ |
| Comparability | Comparability of cases and controls on the basis of the design and analysis | ☆☆ | ☆☆ | ☆☆ | ☆ | ☆ | ☆☆ | ☆☆ | ☆ | ☆ | ☆☆ | ☆☆ | ☆ |
| Exposure | Ascertainment of exposure | ☆ | ☆ | ☆ | ☆ | ☆ | ☆ | ☆ | ☆ | ☆ | ☆ | ☆ | ☆ |
|  | Same method of ascertainment for cases and controls | ☆ | ☆ | ☆ | ☆ | ☆ | ☆ | ☆ | ☆ | ☆ | ☆ | ☆ | ☆ |
|  | Non-Response rate | ☆ | ☆ | ☆ | ☆ | ☆ | ☆ | ☆ | ☆ | ☆ | ☆ | ☆ | ☆ |
| Total scores |  | 8 | 8 | 8 | 7 | 7 | 8 | 8 | 7 | 7 | 8 | 8 | 7 |

Notes: 1. Wang et al. 2018, 2. Fu et al. 2017, 3. Li et al. 2017, 4. Lin et al. 2014, 5. Li et al. 2014, 6. Matsumoto et al. 2013, 7. Junior et al 2003, 8. Xu et al. 2003, 9. Zhang et al. 2003, 10. Peng et al. 2002, 11. Zhang et al. 2001, 12. Liao et al. 1998.
